# Speech and Language Therapy Weekend Service in Inpatient Rehabilitation: A Qualitative Study Exploring Perspectives of People With Stroke

**DOI:** 10.1111/1460-6984.70077

**Published:** 2025-07-03

**Authors:** Lidia Davies, Lucette Lanyon, Robyn O'Halloran

**Affiliations:** ^1^ Discipline of Speech Pathology, School of Allied Health, Human Services and Sport La Trobe University Bundoora Australia; ^2^ Speech Pathology Department Barwon Health Geelong Australia; ^3^ Centre of Research Excellence for Aphasia Recovery and Rehabilitation La Trobe University Bundoora Australia

**Keywords:** patients’ perspectives, qualitative research, rehabilitation, stroke, SLT, weekend service

## Abstract

**Background:**

An allied health weekend service in subacute inpatient stroke rehabilitation can improve patient and organisational outcomes. However, there is insufficient evidence to justify the role of a speech and language therapy (SLT) weekend service in this setting. Exploring the perspectives of individuals who have received SLT services in inpatient stroke rehabilitation will assist with understanding the current role of SLT and the potential for SLT weekend services in inpatient stroke rehabilitation.

**Aims:**

This study aimed to: (1) determine the aspects of an SLT inpatient rehabilitation service that are perceived to be important to people with stroke who have an acquired communication and/or swallowing impairment, and (2) establish which of these valued aspects are also relevant to the provision of an SLT weekend service.

**Methods and Procedures:**

A generic qualitative approach was employed. Adults admitted to inpatient rehabilitation for a minimum 2‐week stay, including two weekends, and who engaged in communication and/or swallowing‐related rehabilitation were invited to participate in semi‐structured, in‐depth interviews. Interview data were recorded, transcribed, and analysed using reflexive thematic analysis.

**Outcomes and Results:**

Six people with stroke were interviewed. Two main themes informed participants’ perspectives of important aspects of inpatient rehabilitation: (1) ‘recovering from my stroke’ which described factors relating to the patients’ participation in their rehabilitation program and their therapy progress, and (2) ‘supporting my wellbeing’ which reflected the value of social connections, feeling supported by staff, dedicated rest periods, and engagement in meaningful activities outside of scheduled therapy sessions. Although some participants supported the idea of an SLT weekend service to receive additional therapy, weekends were also perceived as important to support wellbeing by participating in activities that were not directly part of their rehabilitation program.

**Conclusions and Implications:**

Weekends were perceived to provide both the opportunity for additional therapy and to facilitate valued experiences that went beyond participation in the rehabilitation program. These experiences included rest, maintaining or developing social connections and engagement in social‐based activities. This study's findings have important implications for the development of an SLT weekend service in inpatient stroke rehabilitation.

**WHAT THIS PAPER ADDS:**

*What is already known on the subject*

- Existing evidence on allied health weekend services within inpatient stroke rehabilitation has primarily focused on the outcomes of physical‐based interventions, provided by physiotherapy and /or occupational therapy. The effectiveness of an SLT weekend service in this setting remains unclear. Furthermore, the perspectives of people with lived experience of this service are unknown.

*What this paper adds to existing knowledge*

- This study provides insights into the perspectives of people with acquired communication and/or swallowing impairments post stroke on the role of an SLT weekend service within inpatient stroke rehabilitation. Although weekends were considered to provide an opportunity for the delivery of additional therapy, participants also perceived weekends to play an important role in ensuring rest, facilitating social connections with existing social networks, and participating in meaningful, social activities outside of the therapeutic context.

*What are the potential or actual clinical implications of this work?*

- Participants perceived that weekends were important not only to extend their weekday rehabilitation program but also to facilitate rest, social connections and engagement in socially‐based activities. Understanding the experiences and preferences of people with lived experience of inpatient stroke rehabilitation is essential when planning and implementing an SLT weekend service within an inpatient stroke rehabilitation setting.

## 1 Introduction

Stroke rehabilitation aims to maximise an individual's independence and function (Stroke Foundation 2022b). Within a hospital‐based inpatient setting, it offers intensive, time‐limited and goal‐directed programs delivered by a specialist multidisciplinary team (Stroke Foundation 2022b). Speech and language therapists, as members of this team, provide specialist skills in the assessment and management of communication and swallowing impairments post stroke (Lindsay et al. 2016).

Inpatient stroke rehabilitation is part of subacute care that is provided within Australia's hospital inpatient services (Australian Institute of Health and Welfare 2013). As distinct from acute care and maintenance care, the primary goal of the subacute healthcare team focuses on optimising function and quality of life (Australian Institute of Health and Welfare 2013). The delivery of inpatient stroke rehabilitation in subacute inpatient rehabilitation settings relies on adequate resources and appropriate staffing levels (Stroke Foundation 2022b). An ongoing challenge within subacute care is how to provide the recommended intensity of therapy (Stroke Foundation 2022b). Current evidence indicates that service providers are unable to deliver recommended therapy intensity across a 5‐day week (Intercollegiate Stroke Working Party 2023; Stroke Foundation 2022b), prompting the consideration of weekends as a way to meet these standards (Centres for Medicare and Medicaid Services 2017; Stroke Foundation 2022b).

There is evidence on the effectiveness of a broader allied health weekend service in the subacute inpatient rehabilitation setting. Findings from a systematic review (Sarkies et al. 2018) and additional studies (including Caruana et al. 2017; Caruana et al. 2022; Davidson et al. 2019) support the provision of an allied health weekend service in subacute inpatient rehabilitation across a range of diagnostic groups, including stroke, to improve both clinical and organisational outcomes. However, research to date has centred around physical‐based allied health therapies, specifically physiotherapy and occupational therapy. None of the studies evaluated speech and language therapy (SLT) weekend services.

An exploration of existing SLT weekend inpatient rehabilitation services is in its early stages. Davies et al. (2022) reported findings from an online survey of 83 speech and language therapists on weekend services in inpatient stroke rehabilitation across Australia. More recently, Dunn et al. (2024) surveyed 67 SLT managers or their representatives on SLT weekend services across a broad range of healthcare settings in Australia, including subacute inpatient rehabilitation. Both studies revealed that an SLT weekend service was not standard practice in Australian subacute inpatient rehabilitation facilities. In addition, the clinicians and managers who supported the provision of a weekend service advocated for the service to be an extension of the weekday model of inpatient rehabilitation, one that emphasises therapy instead of a more acute‐type service that prioritises risk minimisation (e.g. dysphagia assessment). Respondents from both studies believed that an SLT weekend service could improve clinical outcomes through increased intensity of therapy. Furthermore, they identified similar barriers to the implementation or continuation of a weekend service, such as insufficient workforce capacity, and concerns that having different clinicians on weekends and weekdays might negatively impact continuity of care, patient outcomes and patient engagement.

Existing qualitative research has explored the experiences and perspectives of patients receiving general inpatient rehabilitation weekend services by allied health professionals more broadly, and has predominantly focused on physical‐based therapies (Gill et al. 2014; Luker et al. 2015; Peiris et al. 2012). Participants in these studies considered allied health weekend service as playing a role in facilitating increased therapy intensity, providing opportunities for social interactions and reducing feelings of boredom.

To date, there are no known studies exploring patients’ perspectives on SLT‐specific weekend services in stroke inpatient rehabilitation. Patient perspectives about healthcare are increasingly considered essential to inform patient‐centred care and the planning, delivery and review of healthcare services (Australian Commission on Safety and Quality in Health Care 2023).

### 1.1 Aims

The aim of this study was to investigate the role of an SLT weekend service within inpatient subacute rehabilitation from the perspective of adults with communication and/or swallowing impairments resulting from stroke. The investigation was guided by two research questions: (1) What aspects of an SLT inpatient rehabilitation service are perceived to be important to people with stroke who have an acquired communication and/or swallowing impairment? and (2) Which of these aspects are important in the provision of an SLT weekend service?

## 2 Methods

### 2.1 Design

A generic qualitative approach was employed to enable a descriptive analysis of human experiences (Caelli et al. 2003; Sandelowski 2000). The Consolidated Criteria for Reporting Qualitative Studies (COREQ) (Tong et al. 2007) guided the conduct and reporting of the study. Ethical approval was granted by the relevant health service (reference number 21/128).

### 2.2 Participant Recruitment

Eligible individuals were at least 18 years of age, had been admitted into an inpatient subacute hospital setting for stroke rehabilitation for a minimum of 2 weeks and two weekends, had received communication and/or swallowing rehabilitation during their admission, were discharged home into the community, and were able to be interviewed in English with or without communication support. Communication supports included modified written information (Rose et al. 2011) and supported conversation strategies (Kagan 1998). A minimum of at least two weekends during admission was required to ensure participants had experience of an inpatient stay on weekends.

Participants were recruited from a single inpatient subacute rehabilitation unit in Victoria, Australia, using a purposive sampling approach. A maximum variation strategy was applied, recognising that experiences and perspectives may vary with different characteristics (Sandelowski 2000) and over time (Kirkevold 2002). Given time and resource constraints, a minimum of six people with stroke were recruited for this exploratory study. Participants were purposively sampled to include at least one participant with: mild communication impairment, severe communication impairment, mild swallowing impairment and severe swallowing impairment. To ensure the data reflected changes in participants’ perceptions regarding the value of inpatient rehabilitation over time, we sought to recruit at least two participants who had been discharged within two months, and two between 6 and 12 months.

The inpatient subacute rehabilitation unit is a 100‐bed facility that provides subacute care across a variety of clinical units, including neuro rehabilitation, geriatric evaluation and management, and orthopaedic rehabilitation. A physiotherapy and occupational therapy weekend service exists across different units, but not SLT.

The first author accessed the health facility's clinical databases and electronic records to identify prospective participants. Communication and/or swallowing impairment severity was determined by reviewing patient medical records for relevant assessment results on admission and then rating communication and/ or swallowing impairment severity using the Australian Therapy Outcome Measures (AusTOMS) Tool (Perry and Skeat 2004). Individuals who met the eligibility criteria and the maximum variation sampling matrix were sent an invitation letter. If no contact was initiated by the prospective participant, they were contacted by a third party (who was not directly involved in the study). Prospective participants who expressed interest received a participant information and consent form (PICF). The first author discussed the details of the study with each prospective participant either face to face or over the phone using communication supports where required in order to optimise the person's ability to understand the conditions of the study (Lanyon et al. 2018; Penn et al. 2009). All written materials used during the recruitment and consent stages were developed in aphasia‐friendly format (Herbert et al. 2019). The combination of simplified written materials and extended verbal discussions was intended to optimise all participants’ understanding of the study (Brady et al. 2013; Nishimura et al. 2013).

### 2.3 Data Collection

Semi‐structured interviews explored each participant's experience of swallowing and/or communication therapy, their views on an SLT service (existing vs. preferred), and the role of an SLT weekend service to address their needs (see Interview Guide in Supporting Appendix A). Interviews were held at a location of the participants’ choice and were video and/or audio recorded. Interviews were transcribed verbatim, initially using an automatic transcription program through Otter.ai software (Liang and Fu 2023). Manual transcriptions were subsequently completed by the first author to ensure transcription accuracy.

### 2.4 Data Analysis

**FIGURE 1:**
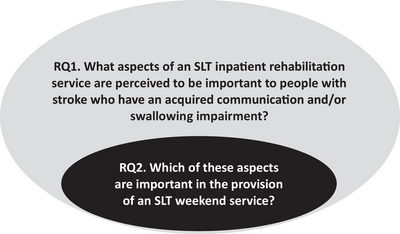
Relationship between the research questions.

Reflexive thematic analysis, as outlined by Braun and Clarke (2022), was undertaken to identify those aspects of an SLT inpatient subacute rehabilitation service perceived to be important to the participants (Research Question 1). The results of this analysis were then used to explore how the provision of an SLT weekend service might contribute to stroke inpatient rehabilitation (Research Question 2). The relationship between these questions is depicted in Figure 1

### 2.5 Rigour

Guba and Lincoln's four principles of rigour in qualitative research—dependability, transferability, confirmability and credibility (Bradshaw et al. 2017)—informed the preparation, implementation and reporting of the study. Dependability strategies included the use of audit trails. To enable readers to judge how applicable or transferable these findings are to their setting, detailed reporting of the sampling process and data collection was undertaken. Measures of confirmability to minimise the impact of bias included thick descriptions supported by sufficient quotes to represent the findings. Lastly, credibility measures included member checking by clarifying and summarising responses with participants at the conclusion of interviews (Lanyon et al. 2018).

## 3 Results

Twenty‐one patients met the eligibility criteria and were sent an invitation letter. Six individuals declined and eight did not reply to the letter and follow‐up phone calls. Seven expressed interest, with six proceeding to the formal consent stage and were subsequently interviewed.

### 3.1 Participant Characteristics

The four men and two women who participated in interviews were aged between 56 and 96 years of age (average 72 years). Two lived alone and four lived with a significant other. No participants engaged in paid employment. Although none of the participants received an SLT weekend service, all participants did receive some physiotherapy and/or occupational therapy weekend service during their inpatient stay. Details of each participant's communication or swallowing impairment and time post‐onset at interview are provided in Table 1.

**TABLE 1:** Participant characteristics (based on maximum variation sampling).

	Gender		Communication impairment: severity		Swallowing impairment: severity		Time post discharge	
	Male	Female	Mild	Severe	Mild	Severe	<6 months	>6 months
PWS1	✓			Aphasia mod‐severe				✓
PWS2	✓		Dysarthria				✓	
PWS3	✓		Dysarthria + dysphonia			Mod‐severe		✓
PWS4		✓	Aphasia mild‐moderate					✓
PWS5	✓		Dysarthria mild‐moderate				✓	
PWS6		✓		Aphasia mod‐severe			✓	
Total	4	2	4	2	0	1	3	3

Five participants were interviewed face‐to‐face and one participant was interviewed over the phone. Among the five face‐to‐face interviews, three were undertaken in the participant's home, and two occurred within a healthcare facility. At the request of the participant, a support person was present in four of the interviews.

### 3.2 Qualitative Data

**Question 1. What Aspects of an SLT Inpatient Rehabilitation Service Are Perceived to be Important to People with Stroke Who Have an Acquired Communication and/or Swallowing Impairment?**

**FIGURE 2:**
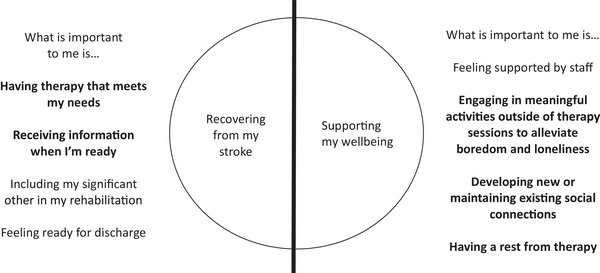
Relationship between RQ1 and RQ2, and their corresponding themes and subthemes.

Thematic analysis resulted in the identification of two main themes that represented aspects of an SLT inpatient rehabilitation service identified as important to the participants: (1) ‘recovering from my stroke’ and (2) ’supporting my wellbeing’. Each of these two themes was informed by four subthemes, as depicted in Figure 2. The sub‐themes are described below, and additional quotes are included in Supporting Appendix B.

#### 3.2.1 Theme: Recovering From My Stroke

Participants valued different aspects of the therapeutic process intended to maximise recovery from their communication and/or swallowing impairments and facilitate their discharge home. These aspects were captured within four subthemes described as: ‘having therapy that meets my needs’, ‘receiving information when I'm ready’, ‘including my significant other in my rehabilitation’ and ‘feeling ready for discharge’.

##### 3.2.1.1 Subtheme: Having Therapy That Meets My Needs

Participants wanted SLT to be meaningful and relevant, and to be based on ‛achievable’ (PwS3) goals. They highlighted the importance of experiencing progress: ‛how much improvement I made [referring to dysphagia therapy] … gave me, great, um, great satisfaction’ (PwS3). Participants valued therapy, which was personally relevant and which facilitated functional improvement: ‛… the practice [referring to therapy targeting speech intelligibility] was very useful, and um, I don't think I'm that hard to understand anymore’ (PwS5). Interesting and varied therapy was also viewed as important: ‛… as long as it's not boring’ (PwS1), with therapy directed by a stable team of skilled staff.

The concept of ‘more is better’ underpinned participants’ perceptions of therapy. More therapy was regarded as a way to increase the rate of recovery: ‛I was … 100 percent keen to … do as much … therapy [in general] … because I wanted to um, well, I wanted to recover’ (PwS3). The provision of regular and frequent therapy was also highlighted as important: ‛I believe that you did need to do it [referring to dysphagia therapy] at least five days a week’ (PwS3).

Participants provided information on preferred formats of therapy. One participant did not welcome the option of independent practice and favoured personalised contact: ‛yeah, much better if you've got somebody's help [referring to aphasia therapy], much better’ (PwS4). One participant preferred one‐on‐one sessions instead of group communication therapy: ‛Oh, for me, I think maybe the one on one [referring to dysarthria therapy] was appropriate for me’ (PwS2).

##### 3.2.1.2 Subtheme: Receiving Information When I'm Ready

Some participants valued receiving education about their stroke: ‛it was so informative and so helpful. I can't put into words how I am, how grateful I was for the girls to um, to help me, to get me, to give the insight into … what had happened to me and why it had happened’ (PwS3). Others described difficulties processing information or coping with the emotional impact from the stroke and preferred postponement of education until after discharge from the unit: ‛I know I had a stroke … but I suppose I didn't really want to know about it’ (PwS4).

Participants valued being informed about their scheduled therapy sessions: ‛in hospital I was moved around quite a lot … whereas here … I knew what to expect’ (PwS5). One participant highlighted the importance of timely and relevant information about their rehabilitation: ‛if you don't know it's [referring to language‐based therapy] there, you can't ask for it’ (PwS3).

##### 3.2.1.3 Subtheme: Including My Significant Other in My Rehabilitation

Participants commented on the role of significant others in terms of their involvement in therapy activities. Significant others were perceived as playing an important part in facilitating the person with a stroke's progress. However, this view was not shared amongst all participants. One individual who experienced a mild dysarthria reported no need for their significant other to be included in therapy sessions: ‛I think, due to the severity of my stroke, I don't think it would have made a difference’ (PwS2).

Participants also valued the role of their significant others in terms of assisting with planning and scheduling community‐based services and local therapy providers: ‛with all the services that I could have, they helped [name of significant other] with all those sorts of things … ’ (PwS4).

##### 3.2.1.4 Subtheme: Feeling Ready for Discharge

Participants appreciated the support they received during their inpatient rehabilitation stay, which facilitated them to feel prepared for discharge. This preparation was partially attributed to their therapy program by reaching a point in their progression where they felt ready for the transition to home. Participants also valued their therapist's efforts in ensuring a seamless and timely transition from inpatient to community‐based therapy: ‛I know that [name of private speech and language therapist] had spoken to um … [name of treating speech and language therapist from the rehabilitation unit] … a couple of times so there was um that continuity’ (PwS5). Furthermore, participants placed importance on knowing that their significant other could support them after discharge.

#### 3.2.2 Theme: Supporting My Wellbeing

Participants identified a range of factors that they perceived as contributing to the functional recovery of their communication and/or swallowing impairment during their admission to the inpatient rehabilitation unit. However, they also valued experiences that extended beyond the direct participation in the rehabilitation program and which appeared to play a critical role in enhancing their overall wellbeing during their inpatient admission. The theme ‘supporting my wellbeing’ resulted in four sub‐themes: ‘feeling supported by staff’, ‘having a rest from therapy’, ‘engaging in meaningful activities outside of therapy sessions to alleviate boredom and loneliness’ and ‘developing new or maintaining existing social connections’.

##### 3.2.2.1 Subtheme: Feeling Supported by Staff

Participants appreciated the support they received from the multidisciplinary team (and not specific to SLT) that contributed to their sense of wellbeing. They highlighted feeling cared for: ‛… everyone cared… that's what I remember’ (PwS4). They appreciated staff's patience: ‛they [team] were excellent … putting up with me’ (PwS1), valued the trust they felt towards their treating team: ‛I knew I was being helped’, and the overall environment within which they spent their inpatient stay: ‛… I really did feel safe there … ’ (PwS4).

##### 3.2.2.2 Subtheme: Having a Rest From Therapy

Some participants placed importance on opportunities to rest from therapy. They looked forward to days without scheduled sessions: ‛… you sort of look forward to um days when you, when you didn't have things to do’ (PwS5). One participant also reported the occasional need for non‐attendance of scheduled weekday therapy sessions in order to rest: ‛there were certain times I had to stop … I felt it was just too much’ (PwS6).

##### 3.2.2.3 Subtheme: Engaging in Meaningful Activities Outside of Therapy Sessions to Alleviate Boredom and Loneliness

Participants conveyed feelings of boredom during their inpatient rehabilitation stay: ‛I was bored, I couldn't read, ah, television, I wasn't interested in [it] … ’ (PwS4). Feelings of loneliness were also identified: ‛I noticed that, um, every other patient had a, had visitors, except for me … which was a little bit sad …’ (PwS3).

Participants described a range of informal activities to address these challenges, including the opportunity to go on day leave—‛I did have the occasional xxx pass, and I got out into the real world’ (PwS2)—or to participate in activities with visitors: ‛… when people came … we'd go for a walk or something like that, just to get out for a little bit’ (PwS4). Some responses reflected self‐initiated tasks to reduce their sense of boredom or engage in relaxation: ‛… go for walks … pull weeds out of the garden’ (PwS2) and ‛TV, I love to watch a lot of TV’ (PwS6).

##### 3.2.2.4 Subtheme: Developing New or Maintaining Existing Social Connections

The absence of, or access to social connections were frequently raised by participants. They welcomed the opportunity to receive visits from family and friends: ‛… I was watching on the calendar, was counting down the days until he [family member] came’ (PwS3). However, they also identified challenges to these opportunities that were either internally driven such as not being emotionally ready for direct contact with family and friends, or externally driven such as COVID‐19 related visitor restrictions: ‛… nothing on the weekend … long [stressed word] weekends … because you couldn't have people to see you’ (PwS4). Participants also valued connections with staff and other inpatients during their rehabilitation admission: ‛… I wandered down to her room or she wandered into mine, we'd just say hello and whatever’ (PwS4).

**Question 2. Which of these Aspects Are Important in the Provision of an SLT Weekend Service?**

The themes and subthemes generated through Research Question 1 guided a second analysis that considered how the provision of an SLT weekend service might contribute to inpatient rehabilitation. Participants’ responses were considered in terms of the two main themes of ‘recovering from my stroke’ and ‘supporting my wellbeing’. Each of the sub‐themes identified in this analysis is described below. Additional quotes are included in Supporting Appendix C. The themes and subthemes that were generated to answer both research questions are depicted in Figure 2.

#### 3.2.3 Theme: Recovering From My Stroke

None of the participants received an SLT weekend service during their inpatient stay in the rehabilitation unit. Their views on this proposed service delivery model were commonly associated with the subtheme ‘having therapy that meets my needs’. The value of receiving information on the weekends was also described. No participants considered an SLT weekend service as an opportunity to include significant others in their rehabilitation or to assist in their preparation for discharge.

##### 3.2.3.1 Subtheme: Having Therapy That Meets My Needs

Participants reported that if they were to engage in therapy on the weekends, these sessions should reflect the weekday program: ‛seven days a week, planning and planning right through’ (PwS1). They also highlighted the importance of therapy being varied and interesting, as this would influence their willingness to participate in weekend therapy: ‛a program around seven days a week … with, with an ‘a la carte’ menu …’ (PwS1). The view of ‘more therapy is better’ also contributed to a positive perception of a weekend service: ‛… put one on one every day … ideally’ (PwS1).

As described earlier, participants valued being treated by the same, familiar team. PwS5 queried the impact of therapy provided on the weekend if led by different or unfamiliar staff: ‛the therapy is more about who um … the therapy was more depending more on who there was, who would come on and on which days … ‘cause you would have different people on different days’ (PwS5).

##### 3.2.3.2 Subtheme: Receiving Information When I'm Ready

Participants did not associate the weekends with an opportunity to receive education on stroke or on their therapy. Instead, the weekends were perceived as an opportunity to process what was learnt in SLT sessions throughout the previous week. Some participants reported that the weekend provided them with the opportunity to orientate themselves for the upcoming week, marking the transition between the previous therapy‐filled week and the beginning of a new one: ‛you didn't have the effect of all the days running together … markers to know um to know what day it was, what to expect’ (PwS5). In this sense, weekends without therapy contributed to feeling oriented and informed.

#### 3.2.4 Theme: Supporting My Wellbeing

In contrast with the theme ‘recovering from my stroke’, where participants’ responses largely centred around one subtheme (‘having therapy that meets my needs’), perspectives that reflected the theme ‘supporting my wellbeing’ represented a range of subthemes.

##### 3.2.4.1 Subtheme: Having a Rest from Therapy

Participants valued days that were dedicated to rest, particularly on the weekends when they did not have scheduled sessions: ‛… you sort of look forward to um days when you, when you didn't have things to do’ (PwS5). They looked forward to these days as an opportunity to recover from the weekdays’ busy scheduled therapy program and challenging sessions: ‛actually one thing I did find was the speech therapy was more tiring than some of the physio ones’ (PwS5).

##### 3.2.4.2 Subtheme: Engaging in Meaningful Activities Outside of Therapy Sessions to Alleviate Boredom and Loneliness

Whilst acknowledging the need for rest, some participants voiced their dissatisfaction with the lack of opportunities to engage in meaningful activities. Feelings of boredom were reported, with little to do in the absence of scheduled therapy: ‛the weekends were quite boring’ (PwS2); and feelings of loneliness, ‛and I would, used to, um, loathe the weekends because um I knew that no one was coming … and I knew that I'd just be sitting in the room just watching that blue wall and the clock … and the chart’ (PwS3).

Agreeing to attend SLT on the weekend was identified as an option for ‛something to do’ (PwS4). In contrast, one participant raised the risk of exacerbating their sense of boredom by attending therapy on the weekends: ‛… looking back on it now, I would say um, if I had it [dysphagia therapy] continued, if I had it on the weekend as well, it could have become quite boring … I'd say I don't think it would, it would have helped … it did become very much same, same, same’ (PwS3). The option of allocating time to social‐based activities on the weekend was appealing to one participant: ‛… that would have been actually very nice’ (PwS3).

##### 3.2.4.3 Subtheme: Developing New or Maintaining Existing Social Connections

Participants regarded the weekends as an important opportunity to receive visitors: ‛… I noticed generally most patients used to um, enjoy the weekend because they knew that there were visitors coming’ (PwS3). Maintaining these existing social connections was often viewed as more valuable than having SLT on weekends. Some participants identified isolation and loneliness without visitors and indicated that they would choose to participate in therapy in order to reduce feelings of isolation:

“… every other patient had … visitors except for me…”(PwS3)

“… are you saying that you would have liked to have had therapy on the weekends?”

“… look, probably yes” (PwS3)

Participants also valued the concept of social activities on the weekends. These activities were considered as opportunities for social interaction and connection with others rather than for formal therapy.

## 4 Discussion

This study explored the perspectives of people with stroke who have an acquired communication and/or swallowing impairment on SLT subacute inpatient rehabilitation weekend services. Data revealed the elements of an SLT rehabilitation service perceived by participants as important. These elements were then considered within the context of application to an SLT weekend service. Although participants were asked questions that were specifically related to SLT, their responses were often not directly related to one discipline. Two distinct themes were identified: ‘recovering from my stroke’ and ‘supporting my wellbeing’.

The first theme reflected participants’ appreciation of the rehabilitation that they received during their admission to the unit. Those aspects that were perceived as important are also reflected in the definition of stroke inpatient rehabilitation as outlined by the Stroke Foundation (2022b). Participants valued goal‐directed, intensive therapy provided by a consistent team of skilled healthcare professionals to assist in a timely and seamless transition back into the community. They also preferred meaningful, varied, and interesting therapy to facilitate their motivation. Furthermore, they welcomed the inclusion of significant others in the therapeutic process when needed. The second theme, ‘supporting my wellbeing’ encompassed factors peripheral to formal therapeutic activities and incorporated aspects that improved participants’ overall inpatient experience. These factors included the emotional support received from healthcare staff, the importance of connecting with their existing social networks as well as the opportunity to create new ones, designated rest periods, and engagement in meaningful activities.

Findings from previous studies that have described patients’ experiences during their admission to an inpatient rehabilitation unit are consistent with those from our study. They include two systematic reviews exploring the experiences and perspectives of people with stroke on the rehabilitation that they received (Luker et al. 2015; Peoples et al. 2011). The reviews highlighted the importance that participants placed on receiving information to enable self‐advocacy in the development of their rehabilitation program, on engaging in interesting therapy activities, and on receiving high‐intensity intervention. Additionally, the reviews revealed experiences of boredom and loneliness, the value of social‐based activities, the need for rest, and the importance of their relationships with and support from staff. Our current study also identified the reliance on significant others as part of their rehabilitation journey within the inpatient rehabilitation setting, which was not highlighted in the findings from the two systematic reviews.

The perceived need for weekends to address wellbeing underscores the importance of personally tailored patient‐centred care. This view differs from findings from the recent surveys of Australian speech and language therapists (Davies et al. 2022) and SLT managers (Dunn et al. 2024). A perception commonly shared by respondents from these studies described the ideal SLT weekend service within stroke inpatient rehabilitation as an extension of the weekday service, combining assessment, therapy, education and training. Although these aspects may be important, an understanding of patient perspectives and how/if an SLT weekend service can contribute to their wellbeing outside of formalised therapy sessions also requires consideration.

In considering the potential delivery of SLT on the weekends, some participants were supportive of a weekend service in situations where the therapy was interesting, enjoyable and personally relevant to them. Participants welcomed the idea of a weekend service, guided by their perception that increased therapy intensity would enable quicker attainment of goals and an earlier discharge. The benefit of increased therapy intensity in SLT were also perceived by SLT clinicians and managers in earlier studies (Davies et al. 2022; Dunn et al. 2024). It is unclear to what degree these beliefs are supported by research evidence and clinical guidelines. For example, stroke clinical guidelines across four western countries have recommended at least 3 h of therapy from a multidisciplinary team (including SLT) per day for at least 5 days a week (Australasian Faculty of Rehabilitation Medicine 2019; Intercollegiate Stroke Working Party 2023; National Institute for Health and Care Excellence 2023; Teasell et al. 2020; Winstein et al. 2016), or a minimum of 15 h over the 7 day week (Centres for Medicare and Medicaid Services 2017). In terms of an SLT service, a range of recommended level of therapy intensity exists internationally. The stroke clinical guidelines from the United Kingdom and Ireland recommend the provision of at least 45 min of SLT a day, 7 days a week, and as much aphasia therapy as possible until progress plateaus (Intercollegiate Stroke Working Party 2023). In contrast, 30–45 min, 2–3 days per week of aphasia therapy within the first 6 weeks post stroke onset is recommended in Australia (Stroke Foundation 2022a). The provision of at least 5 days a week of dysphagia rehabilitation is recommended in the United Kingdom (National Institute for Health and Care Excellence 2023) whereas the World Stroke Organisation advocates for the provision of dysphagia education and rehabilitation and the facilitation of compensatory strategies at least three times a week (Mead et al. 2023). Effectiveness of treatments for specific swallowing and communication disorders is less clear. Most research to date has investigated aphasia therapy, with current evidence supporting frequent SLT of 3–5 or more days a week (The REhabilitation and recovery of peopLE with Aphasia after StrokE [RELEASE] Collaborators 2022).

Staffing models were identified in the interviews as a potential barrier for the delivery of a quality weekend service. One participant (PwS5) queried the effectiveness of a weekend service if personnel were not part of the weekday treating team, suggesting the therapeutic relationship that builds over time between clinicians and patients is important to the recovery process. This finding was consistent with clinician‐reported concerns that different staff on the weekends may impact clinical outcomes, patient engagement and continuity of care (Davies et al. 2022).

The weekends may play a role in the provision of education and training for significant others rather than inpatients themselves. For some people with stroke who participated in the interviews, receiving comprehensive education within their inpatient admission was highly valued, whilst others preferred to have it postponed until after discharge. Participants did not support the allocation of weekends for the provision of education and training. A weekend service may be more appropriate for the provision of education and training of significant others instead of people with a stroke. Cameron et al. (2013) explored the perspectives of significant others across the continuum of care. The authors highlighted participants’ frustration with the lack of access to the multidisciplinary team for the purpose of education and training within the inpatient stroke rehabilitation setting. The authors suggested the provision of a weekend service to address this perceived gap. The opportunity to use weekends as a means to provide education and training to significant others, as well as enabling them to be involved in therapy activities, was addressed in an editorial by Wade (2020).

### 4.1 Limitations

The findings of this study provide a valuable albeit preliminary exploration of the perceptions of people with stroke on an SLT weekend service in the subacute inpatient rehabilitation setting. Participants were recruited from one rehabilitation unit within a regional area. It is possible that the experiences, needs, and preferences of stroke survivors who received sub‐acute inpatient stroke rehabilitation in other healthcare organisations in metropolitan, rural or other regional locations may generate different and novel insights.

Efforts were made to describe a broad range of perspectives through the use of maximum variation sampling. However, the majority of participants presented with communication impairments, and only one with dysphagia. The findings of the study may have been influenced by the participants’ profiles.

Due to resource limitations, stroke survivors who required an interpreter could not be included in the current study. In addition, stroke survivors who were discharged to residential care or supported accommodation were not interviewed. People who are discharged from inpatient stroke rehabilitation to residential care or supported accommodation are likely to have very different experiences and perspectives about stroke inpatient rehabilitation compared with individuals who are discharged to their home in the community. The study's findings should be considered within the context of these limitations.

Finally, none of the participants in this current study had received an SLT weekend service during their admission to the rehabilitation unit. It is unclear whether their views would be different if they had received a weekend SLT service. Interestingly, Peiris et al. (2012) compared the perspectives of patients who had and had not participated in a weekend physiotherapy service. They noted that perspectives differed significantly between the two groups. Those patients who had participated in therapy on Saturdays viewed weekends as a means for additional rehabilitation, whilst those who did not perceived the weekends as a source of rest.

### 4.2 Implications for Clinical Practice and Policy

This study provides valuable insights into patient perspectives on an SLT weekend service. As noted above, many of the preferences expressed by participants in this current study have also been identified in previous qualitative studies on patient preferences relating to physical therapies in post‐stroke inpatient rehabilitation. This suggests that the findings identified here can be integrated into the broader planning of inpatient allied health weekend rehabilitation services.

With increasing pressure to reduce length of stay in inpatient rehabilitation facilities whilst optimising patient experience, the healthcare team must optimise the limited time they have with patients. This responsibility could extend beyond individual therapy sessions to consider the patients’ experience more broadly. The current study highlights the diversity in participants’ experiences, needs and preferences in relation to SLT weekend service in stroke inpatient rehabilitation. Results support the need for flexibility and a patient‐centred approach when considering the role of the SLT weekend service in stroke rehabilitation. Lastly, allied health weekend services might involve an integrated multidisciplinary approach that addresses therapeutic goals and/or patient wellbeing using socially oriented activities. SLT could have a critical role in ensuring that patients with communication and swallowing difficulties are included as fully as possible in these activities.

### 4.3 Future Directions

Research into SLT weekend service in inpatient stroke rehabilitation is in its preliminary stages. Further in‐depth exploration into the perspectives of key stakeholders positioned within the subacute inpatient stroke rehabilitation setting could inform the generation of future services. Healthcare staff, such as SLT managers responsible for service delivery, and the broader multidisciplinary team, including speech and language therapists working in this setting, would provide invaluable insights. Tapping into perspectives of people with stroke from different contexts, such as metropolitan and regional health networks, encompassing individuals who have received an SLT weekend service, or who have been discharged to various settings, would enable novel understandings into the diverse needs and preferences of this population. In addition, research into the perspectives of their significant others is recommended. Furthermore, the authors acknowledge the importance of quantitative‐based research, investigating the effectiveness of an SLT weekend service in stroke inpatient rehabilitation across clinical, organisational and health‐related quality of life outcomes.

## 5 Conclusion

People with stroke who received SLT‐based therapy for their communication and/or swallowing difficulties in an inpatient rehabilitation setting valued activities that supported their recovery from stroke and their wellbeing. Although participants perceived the benefits of continued therapy on the weekend, they also wanted dedicated time to support their wellbeing. The valuable insights generated from this study could inform the future development of an SLT weekend service within inpatient stroke rehabilitation.

## Ethics Statement

The ethics committees from both Barwon Health and La Trobe University granted ethical approval for the study. The National Statement on Ethical Conduct in Human Research (National Health and Medical Research Council, 2018) guided the implementation of measures to protect participants’ privacy.

## Consent

All participants provided consent. Where written consent was not feasible, verbal consent was audio‐recorded.

## Conflicts of Interest

The authors declare no conflicts of interest.

## Supporting information




**Supporting Appendix A**: Interview Guide
**Supporting Appendix B**: Themes, Subthemes and Example Responses for Research Question 1
**Supporting Appendix C**: Themes, Subthemes and Example Responses for Research Question 2.

## Data Availability

Direct quotations from participants have been used to support the study's analysis. De‐identification of data was undertaken to ensure participants’ confidentiality.
