# Supplementary material for: Speech and Language Therapy Weekend Service in Inpatient Rehabilitation: A Qualitative Study Exploring Perspectives of People With Stroke
Source: Int J Lang Commun Disord. 2025 Jul 3;60(4):e70077. doi: 10.1111/1460-6984.70077 (PMC12223928; doi:10.1111/1460-6984.70077)
Supplement: Supplementary file 1 — Supporting Appendix A: Interview Guide Supporting Appendix B: Themes, Subthemes and Example Responses for Research Question 1 Supporting Appendix C: Themes, Subthemes and Example Responses for Research Question 2. [file JLCD-60-0-s001.docx]

# Appendix A: Interview Guide

| **INTRODUCTORY QUESTIONS (BRIEF)**  **□ I understand that you had a stroke and you were admitted to IRC. What was that like?**  *Prompts (if needed):*   - *Tx in gym, kitchen area, groups / tx on arms, legs, swallowing. / physio, OT, etc*   **GENERAL EXPERIENCE OF REHAB TARGETING SWALLOWING AND/OR COMM DIFFICULTIES**  **□ What was your communication like after your stroke?**  *Prompts:*   - *Speaking, understanding, reading, writing.* - *Experiences with these difficulties*   **□ Did you have therapy for the communication changes (if relevant)? Tell me about that.**  *Prompts:*   - *What did tx involve? Tasks? In room? Outside room? In groups? With family?* - *What were the effects of these tasks? Did they achieve your goals?*   **□ What was your eating and drinking like after your stroke?**  *Prompts:*   - *Eating/chewing, drinking, F&F going down the wrong way* - *X-ray to test swallow*   **□ Did you have therapy for the changes to your eating and drinking (if relevant)? Tell me about that.**  *Prompts*   - *What did tx involve? Tasks? Where?* - *What were the effects of these tasks? Did they achieve your goals?*   **PERSPECTIVES ON THIS SERVICE PROVISION: WHAT PARTICIPANTS RECEIVED VERSES WHAT PARTICIPANTS WANTED (IN TERMS ON SWALLOWING AND/OR COMMUNICATION THERAPY)**  **□ What did you think about the therapy you got for your communication / eating and drinking?**  *Prompts:*   - *What did you want from the service? Did you get it?* - *What did you get that you did NOT want? Why?* - *What were your goals? Were they addressed? Were they reached?* - *Were you prepared for discharge?*   **□ You have been at home for a while now. Think of what you do now. What kind of things were needed to help you prepare for home in terms of your swallow and/or communication?**  *Prompts:*   - *Education and training? Resources? Practice in the community?* - *Others?*   **Did that happen? What was missing?**  **ROLE OF A SPEECH PATHOLOGY WEEKEND SERVICE IN MEETING PERCEIVED NEEDS AND PREFERENCES**  **□ Did you get any therapy on your communication and/or swallowing on the weekends?**  **If yes: tell me about it.**  *Prompts:*   - *What did it focus on? What did you think about it?*   ***Would you have liked more or it / less of it / the same? Why?***  *Prompts:*   - *More therapy? Opportunity to practice learnt skills in community?* - *Sessions with family / significant other? Education and training?* - *Prefer weekend for rest? Prefer weekend just for visitors not therapy)?*   **If not: Would you have wanted it? Why? Why not?**  *Prompts:*   - *More therapy? Opportunity to practice learnt skills in community?* - *Sessions with family / significant other? Education and training?* - *Prefer weekend for rest? Prefer weekend just for visitors not therapy?*   **CONCLUSION**  **□ Is there anything else you would like to share about your experience of speech therapy at the unit?** |
| --- |

# Appendix B: Themes, Subthemes and Example Responses for Research Question 1

*Table 1: Themes, Subthemes, and Example Responses for Research Question 1 [What Aspects of an SLT Inpatient Rehabilitation Service are Perceived to be Important to People with Stroke Who Have an Acquired Communication and/or Swallowing Impairment?]*

| **Theme** | **Subtheme** | **Example response** |
| --- | --- | --- |
| Recovering from my stroke | Having therapy that meets my needs (therapy that is goal-directed, or varied and interesting; more is better; familiar team of skilled staff) | “…what they, what I did or what they did, is, um, set yourself a goal, and then move, move forward towards that goal, and then achieve that goal, then set yourself another achievable goal” PwS3  “…um, look, you know, I,I,I I believe that not doing one thing all the time, you know, make it varied…just, as long as it’s not boring” PwS1  “um, people used to look at my board and say, ‘Gee, you’re busy…you do a lot of stuff’, but I’d say, ‘I’m glad’. I mean I’d hate to have the, the time just to sit around and waste, I,I,I was really, um, 100 percent keen to um, get out there and do as much physiotherapy and whatever sort of therapy it was I was very keen to do, because I wanted to um, well, I wanted to recover” PwS3  “there was more flow, more continuity” PwS5  “here?” interviewer  “yeah, yeah” PwS5  “…so when you talk about continuity of staff, are you talking about the nursing staff, or are you talking about the therapy staff or everybody?” interviewer  “well, mainly the nursing staff, um…but, um, it was pretty much with everyone else” PwS5 |
|  | Receiving information when I’m ready (being informed on scheduled therapy sessions, therapy approaches or effects of stroke; timing of education) | “you just sort of knew what, what time, sort of, um, you were more aware of what time things came on, and when and how it worked” PwS5  “they told me every time exactly what they were doing and why they were doing it and everything else…I’m one of those people that likes to know” PwS1  “…did you get information then about what had happened to you, and what kind of communication difficulties you had?” interviewer  “not that I can remember” PwS4  “okay, would that have been something you wanted at that stage?” interviewer  “I don’t think so…I just wanted to sort it out” PwS4  “…when I first went there, we all, everyone was in the room and they talked about stroke but I got nothing out of it…I really couldn’t quite go with it” PwS4  “is it because of the communication, you didn’t, you had trouble understanding what they were saying, or was it because you were just overwhelmed?’ interviewer  “overwhelmed probably” PwS4 |
|  | Including my significant other in my rehabilitation (access to education; preparation for discharge; involvement in therapy, depending on patient’s circumstances and impairment severity) | “was there anything else that they gave you, that assisted once you came home?” interviewer  “no, not really, except that, with all the services that I could have, they helped (name of significant other) with all those sorts of things” PwS4  “…and did any of the therapy sessions involve your family or friends and practicing your talking with them?” interviewer  “no” PwS2  “no…so is that something that you think, in hindsight, would you have liked? Would it have been helpful?” interviewer  “um, I think, due to the severity of my stroke, I don’t think it would have made a difference” PwS2 |
|  | Feeling ready for discharge  (progress in therapy to the point of feeling ready for discharge; timely transition from inpatient to community-based therapy; knowing that my significant other can support me when needed) | “they prepared me well enough to go home” PwS1  “…I’m thinking also about the speech therapy that you got at (name of inpatient rehabilitation unit), compared to what you’ve got at (name of community rehabilitation centre)…was there a good um flow?” interviewer  “yes, there was…I felt like I was talking to the same girls…they were very good” (PwS4)  “they made the family aware of what I had to do” PwS1 |
| Supporting my wellbeing | Feeling supported by staff  (feeling cared for; responsive to my needs; feeling safe and secure) | “they gave me a little note [aphasia community card] to give to people…yes, fabulous, I’ve used that several times ... I think that’s great, that’s wonderful, and see, this is the care that I got…and everyone cared” PwS4  “I really did, I really did feel safe there, I knew that I was being helped” PwS4  “this lady [pantry staff] xxx but um, xxx used to know what you wanted, and they would have in their hand before you even asked for it” PwS5 |
|  | Having a rest from therapy  (looking forward to days without scheduled sessions, time out from scheduled sessions) | “yeah, and you um, you sort of look forward to um days when you, when you didn’t have things to do” PwS5  “and there was just sometimes, I felt that I had to rest” PwS6 |
|  | Engaging in meaningful activities outside of therapy sessions to alleviate boredom and loneliness | “just being in the same room, as I said, twenty four seven was just so frustrating” PwS3  “what kind of things did you do on the weekend?” interviewer  “go for walks…exercises…pull weeds out of the garden [laughing]” PwS2  “in the rehab centre? Did you? [laughing]” interviewer  “I was bored out of my brain” PwS2 |
|  | Developing new or maintaining existing social connections (connections with family, friends, staff, other inpatients on ward) | “oh, ah, bit sad, but um, my (significant other) and my (family member) came to see me once on the weekend” PwS3  “it [Breakfast Group] was good to do, rather than just sit…sit on the edge of the bed and wait for people to come to you…um, it was valuable…the therapy sessions of making the breakfast and eating it…and it was with other people” PwS2 |

# Appendix C: Themes, Subthemes and Example Responses for Research Question 2.

*Table 2: Themes, Subthemes, and Example Responses for Research Question 2 [Which of these aspects are important in the provision of a speech pathology weekend service?]*

| **Theme** | **Subtheme** | **Example response** |
| --- | --- | --- |
| Recovering from my stroke | Having therapy that meets my needs (goal-directed therapy to facilitate progress; varied and interesting therapy would influence decision to participate in weekend therapy; more is better; query impact of weekend therapy led by unfamiliar staff) | “and that ideal situation would be…?” interviewer  “seven days a week, planning and planning right through, you know” PwS1  “are you saying then that you would have liked to have had therapy on the weekends?” interviewer  “well, look, it would have, it would have…look, probably yes, but look, it was, it was um, in some ways it was um, it was nice to have a day off…from therapies because, as I said, I was very, um, very, look. I did look forward to them, and I did enjoy them, um, but look…it xxx if you just keep continually doing things day after day, it can get quite mundane, and it was nice to have a day off” PwS3  “if, if, if we had all the staff and all the money in the world, you’d, you’d have ah an ideal situation but…” PwS1  “and that ideal situation would be…?...” interviewer  “seven days a week” PwS1  “and in your personal circumstances … if you have been offered therapy on the Saturday and Sunday, would you have …? Interviewer  “in the early days, I would’ve taken it” PwS1  “okay, and then with regards to the speech therapy, so if it was offered on Saturday and you said ‘yep’, what would that have looked like? So, would that have been the same as the weekday or would you have potentially liked it to look something different?” interviewer  “um, well it was um [pause], bit hard to imaging but um, the therapy is more about who um…the therapy was more depending more on who there was, who would come on and on which days…‘cause you would have different people on different days” PwS5 |
|  | Receiving information when I’m ready (opportunity to orient self for the upcoming week) | “it was quite helpful, I mean um, it also made um, xxx the amount of um progress, you didn’t have the effect of all the days running together…getting into a routine, xxx markers to know um to know what day it was, what to expect…” PwS5 |
| Supporting my wellbeing | Having a rest from therapy | “…it xxx if you just keep continually doing things day after day, it can get quite mundane, and it was nice to have a day off” PwS3 |
|  | Engaging in meaningful activities outside of therapy sessions to alleviate boredom and loneliness (accepting therapy on weekend for something to do) | “…would you have liked therapy on the weekends?” interviewer  “well it would have given me something to do, wouldn’t it?” PwS4 |
|  | Developing new or maintaining existing social connections  (receiving visitors with family or friends seen as more important than having therapy on the weekend; would choose to participate in therapy on weekends in order to reduce feelings of isolation; value social-based activities on the weekends | “would you have liked to have it [therapy] on Saturday and Sunday?” interviewer  “no, not really [laughing]” PwS6  “why is that?” interviewer  “oh, I don’t know, I sort of feel at home Saturday and Sunday…the other days were alright” PwS6  “are you saying…that it was better for you…to have therapy on the weekdays?” interviewer  “yeah, yeah” PwS6  “and the weekend, no therapy?” interviewer  “yeah” PwS6  “and so what would you, what do you think your Saturdays and Sundays are for?” interviewer  “Oh, I don’t know, [laughing] it was just Saturday and Sunday” PwS6  “are they for sleep? Or are they just to watch TV?” interviewer  “yeah, watch TV” PwS6  “or is it for, for visitors? Or is it just to have a rest? interviewer  “well, visitors” PwS6  “visitors?” interviewer  “yeah” PwS6  “and I noticed generally most patients used to um, enjoy the weekend because they knew that that there visitors coming…and I would, used to, um, loathe the weekends because um I knew that no one was coming…and I knew that I’d just be sitting in the room just watching that blue wall and the clock” PwS3  “so are you saying (name of PwS), that you know, potentially, you would have liked something on the weekend, but it might have been something a bit different, so maybe more social or, or something just a bit more interesting?” interviewer  “yeah, that would have been, yeah, that’s, I sort of didn’t look at it like that, that, that would have been actually very nice” PwS3 |
